# Appendicular Lean Mass Index Using Dual-Energy X-Ray Absorptiometry (DEXA) in Professional Football: A Pilot Study of a New Method for Improved Inter-Operator Reproducibility and Analysis of Pelvi-Trochanteric Muscles

**DOI:** 10.3390/sports13090285

**Published:** 2025-08-25

**Authors:** Charles Evrard, Julien Blaess, Thibaut Goetsch, Etienne Fellous, Francois Pietra, Alain Meyer, Margherita Giannini, Bernard Geny

**Affiliations:** 1 Department of Physiology and Functional Exploration, Strasbourg University Hospital, 67091 Strasbourg, France; evrard.charles@outlook.com (C.E.); julien.blaess@chru-strasbourg.fr (J.B.); etienne.fellous@chru-strasbourg.fr (E.F.); alain.meyer1@chru-strasbourg.fr (A.M.); margherita.giannini@chru-strasbourg.fr (M.G.); 2 Biomedicine Research Center of Strasbourg (CRBS), UR 3072, Mitochondria, Oxidative Stress and Muscle Plasticity, Faculty of Medicine, University of Strasbourg, 67000 Strasbourg, France; 3 Department of Public Health, Strasbourg University Hospital, 67091 Strasbourg, France; thibaut.goetsch@chru-strasbourg.fr; 4 Racing Club de Strasbourg Alsace, 67000 Strasbourg, France; medical@rcstrasbourg.eu

**Keywords:** body composition, muscle mass, dual-energy X-ray absorptiometry (DEXA), appendicular lean mass index (ALMI), soccer athletes

## Abstract

**Background:** Body composition assessment is important in professional football as a measure of overall adaptation of the athlete to the training demand and calorie intake. However, it is operator-dependent, relying on subject positioning and the focus angle of the X-rays. In addition, the usual appendicular lean mass index (ALMI) does not include the pelvitrochanteric muscles, which are often implicated in athlete injuries. **Methods:** Three independent operators compared the reproducibility of dual-energy X-ray absorptiometry (DEXA) including pelvi-trochanteric muscle mass in twenty professional football players, using the standard and a new method. **Results:** Mean age, weight, and height of the footballers were 25.9 ± 4.9 years, 79.4 ± 9.4 kg, and 1.83 ± 0.09 m. Using the standard method, the ALMI was 9.28 ± 0.62, 9.20 ± 0.65, and 9.13 ± 0.64 kg/m^2^ for the first, second and third operator, respectively. When including the pelvi-trochanteric muscles, the ALMI values were 11.90 ± 0.66, 11.84 ± 0.63, and 11.83 ± 0.65 kg/m^2^ for the three operators. The difference between the two methods was significant (*p* < 0.001). The mean inter-operator difference was similar regardless of the method used (0.099 ± 0.06 kg/m^2^). The intraclass correlation coefficients (ICC) (A,1) were 0.949 [0.881; 0.979] for the standard method and 0.977 [0.951; 0.990] for the new method. The ICC (C,1) was 0.960 [0.918; 0.983] for the standard method and 0.979 [0.957; 0.991] for the new method. **Conclusions:** Thus, both new and conventional methods showed excellent reproducibility. However, reproducibility and inter-operator variability were better with the adjustment of the new scan lines. Moreover, the inclusion of a larger gluteal and adductors muscle mass was easy to achieve, providing additional information that could potentially be useful for early diagnosis and/or prevention of future muscular injuries in elite athletes.

## 1. Introduction

**Figure 1:**
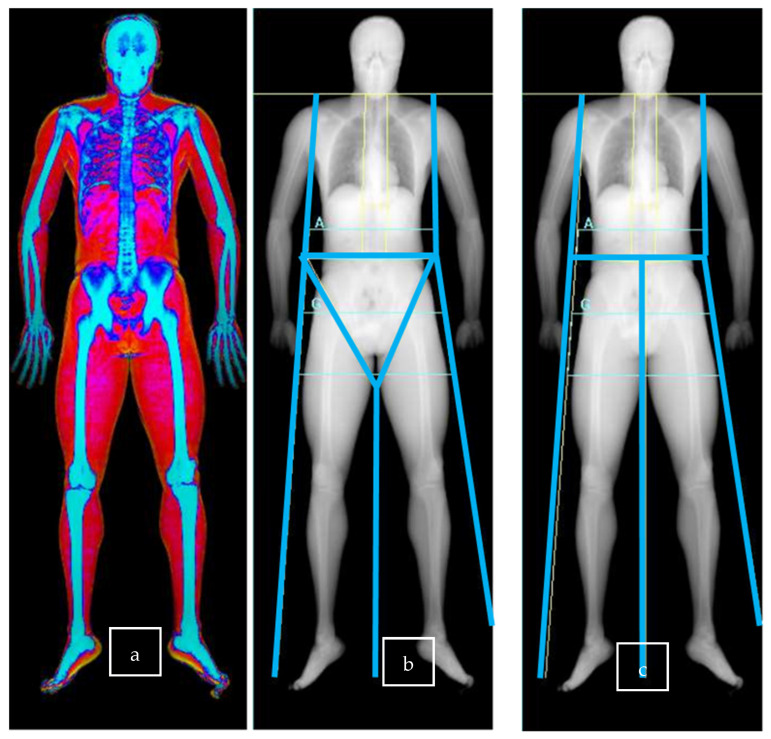
Representation of DEXA imaging with different regions of interest defined by scan line placement. (**a**): Whole-body DEXA. (**b**): Conventional measure scan lines. (**c**): New measure scan lines. A: Androïd area; G: Gynoïd Area.

An athlete’s body composition can be assessed using different methods such as anthropometry (skin fold), body impedance analysis, or dual energy X-ray absorptiometry (DEXA) [1]. Whole-body DEXA (Figure 1a) is a well-known technique for assessing body composition, and its use among athletes has increased in recent years, becoming one of the methods of choice [2]. DEXA allows for three compartment measurement of body composition (fat mass, lean mass, and bone mineral content) with a low radiation dose [3,4,5].

Nowadays, monitoring body composition is important for most professional athletes or teams, as it can change throughout a sport season (pre-season, competition time, post-season) [6,7]. Therefore, monitoring body composition as accurately as possible, appears essential for adapting training load and physical preparation [8,9]. Further, it is well known that body composition and lean and fat mass influence physical performance and injury risk in athletes [10,11,12,13,14,15]. From this perspective, DEXA is one of the most accurate methods for exploring body composition, enabling the monitoring of both athletes and patients. In addition to the direct measurement of total fat, lean, and bone mass, DEXA allows for precise segmentation through the analysis of specific regions of interest (ROIs) [1,16,17,18,19,20,21,22,23,24,25,26,27,28,29].

Nevertheless, DEXA is described as an operator-dependent examination [17,30]. The focal angle of the X-rays has an impact on the assessment of lean, fat, and bone mass, which may be over- or underestimated. During positioning, the operator must ensure that there is no overlap of limb segments. Manual delineation of regions of interest has also been described as a potential source of error due to virtual addition or removal of mass from one compartment to the detriment of another [23]. The main sources of human error are the positioning of the subject before the acquisition phase and the manual demarcation lines of the region of interest (ROI) during the analysis phase [24,25,26,27]. Highlighting the importance of reproducible measurement techniques in sports science applications, best practice protocols, including positioning on the DXA scanning bed, as well as technician’s activities in scan capture, analysis, and interpretation, are now widely proposed, and standardization of DEXA protocols for athletes has recently been highlighted, particularly to enable longitudinal monitoring of body composition and injury [2,17,23,30,31,32,33].

Furthermore, the determination of the appendicular lean mass index is not entirely satisfactory, as current methods exclude a significant pelvic muscle mass due to the positioning of the ROIs [2], although it seems preferable to analyze a muscle group as a whole [34] and despite the importance of these muscles in the movements of athletes [35,36]. The delineation of this region corresponds to an isosceles triangle whose base is a line passing through the upper end of the iliac crests, while the sides are lines running along the lateral edge of the iliac bones and through the center of the femoral necks. This region includes the origin of various muscles [37] positioned anteriorly (psoas major, iliacus, ilio-psoas, quadratus femoris, obtrator externus, adductor magnus, adductor longus, adductor brevis, gracilis, pectineus) and posteriorly (gluteus maximus, superior gemellus, inferior gemullus, adductor magnus, semitendinosus, longum musculi bicipitis femoris, semimembranosus, gluteus medius, tensor facia latae, satorius, rectus femoris).

However, these muscles end outside the DEXA-defined “hip” region, i.e., below the line cutting the center of the femoral neck [2,37] and, importantly, are involved in the various hip movements [38]. For example, flexion requires the activity of iliopsoas (iliacus, psoas major, psoas minor), rectus femoris, tensor fascia latae, and Sartorius) muscles. Extension requires the activity of gluteus maximus, becips femoris, semimembranosus, and semitendinosus. Abduction involves the gluteus medius, gluteus minimus, and tensor fascia latae muscles. Adduction requires the activity of the adductor magnus, longus and brevis. Internal rotation (gluteus medius, gluteus minimus, and tensor fascia latae) and external rotation (obturator internus, obturator externus, superior gemellus, inferior gemullus, piriformis, and quadratus femoris) also involve many muscles.

During walking and running, hip mobility alternates between flexion and extension in the sagittal plane and abduction and adduction in the coronal plane [38]. There is therefore a discrepancy in the DEXA, which classifies in the hip region muscles that may originate in the pelvis but are functionally involved in lower limb activity. This is corroborated by a study of surface electromyography of the adductor magnus, adductor longus, and vastus lateralis during ball striking and single-leg stance, suggesting that the hip adductors are engaged during both the striking and single-leg stance phases [2,37,38,39].

These pelvi-trochanteric muscles are very important in athlete—and can be accurately measured by DEXA [35,36,40,41]—especially soccer players, as they allow the external rotation of the hips. This action is involved during rapid changes in direction, requiring combined activity between these muscles and eccentric contraction of the internal rotator muscles of the hip, including the adductors. The high mechanical demands placed on these muscles during sudden accelerations, decelerations, and change-of-direction maneuvers may contribute to their susceptibility to painful groin injuries, since muscle strength is correlated with muscle mass. The most common injury observed at the 2014 FIFA World Cup was the thigh strain, with a high recurrence rate (approximately 18%) for adductor strains in professional soccer players [42,43,44].

The objective of this study was therefore to determine the feasibility of new adjustments of the scan lines during DEXA, aiming to include greater gluteal and adductors muscle mass in professional soccer players. Indeed, these muscles are widely involved in hip and lower limb biomechanics as well as in soccer player injuries. We also examined whether the modifications of the lower limb ROI, combined with the addition of more precise demarcation lines, improved inter-operator reproducibility, compared to the standard method.

## 2. Methods

### 2.1. Population

Acquisitions were made on June 2022 during a pre-season gathering. We recruited twenty professional footballers from a French football team (Racing Club de Strasbourg Alsace), playing in the first division. All subjects were healthy and informed of the risks and benefits of the study before giving their written informed consent to participate. The study was conducted in accordance with the Declaration of Helsinki and approved by the Ethics Committee of the Faculties of Medicine, Dentistry, Pharmacy, Nursing Schools, Physiotherapy, Midwifery and Hospitals of Strasbourg (CE-2023-71, 7 October 2023).

Body weight was measured with a scale, barefoot and minimally clothed, to the nearest 0.1 kg (Seca 878 Dr, Seca, Hamburg, Germany), and height was measured to the nearest 1 cm.

### 2.2. Methods

#### 2.2.1. General Study Design

The reproducibility of ALMI assessment and segmental leg measures was compared using two methods: standard and new. All subjects fasted overnight, and each served as his control. Subjects underwent whole-body DEXA imaging sessions, according to the manufacturer’s recommended procedures on a Horizon Wi (S/N 303678M HOLOGIC^®^) densitometer (HOLOGIC^®^, Waltham, MA, USA) at standard fan-beam scanning speed. Specifically, DEXA imaging involves two steps: data acquisition, followed by analysis, during which the operator defines the area of interest. This second step allowed for a comparison between the standard and the new method. Before the session, a stepped phantom of six acrylic and aluminum fields of varying thicknesses and known absorptive properties was scanned to serve as external reference for the analysis of various tissue components. Then, an experimented operator placed the subject in a supine position on the scanning table, minimally clothed, and after removing any metallic objects.

After the whole-body DEXA scan (Figure 1a), the software automatically defined the region of interest (ROI) based on different scan lines. Then, if necessary, an experienced operator manually adjusted the ROIs. The leg and arm scan lines are used for ALMI measurement [24,25].

#### 2.2.2. Description of the Digital Scan Analysis Methods

The first, standard method, used the conventional positioning of arms and legs ROIs [17,23]. The scan line separating half-pelvic and ipsi-lateral leg may pass through the center of the femoral neck.

Figure 1b shows the different lines in conventional measure in more detail, as described by Bazzochi et al. [17] and Libber [23].

- The trunk lines: two vertical lines, one for each side, must be placed around the chest/abdomen and separate the arm ROIs from the trunk ROI. The upper portion of each line is called a shoulder cut line, and it has to bisect the humeral-scapula joint.
- The pelvic line: one horizontal line must be placed just above the upper boundaries of the iliac crests.
- The groin lines: two angled lines, one for each side, have to pass through the center of the femoral neck, and a leg line (one vertical line) has to evenly divide the legs and the feet [17].
- The leg line: one vertical line starts at the end of the groin lines and separates the two lower limbs.

The second, new method, the Strasbourg method, aimed to merge the conventional leg and half-pelvis ROIs into a single ROI for each side of the body. Our approach relies on anatomical structures (iliac crests and pubic syndesmosis) that are easier to define.

Figure 1c illustrates the news method in more detail:

- The two trunk lines and the pelvic line were placed with the same landmarks as in the conventional measure.
- The two groin lines were merged with the pelvic line.
- The vertical leg line has to pass through the symphysis and separate, as in the conventional measure, the two lower limbs.

**Figure 2:**
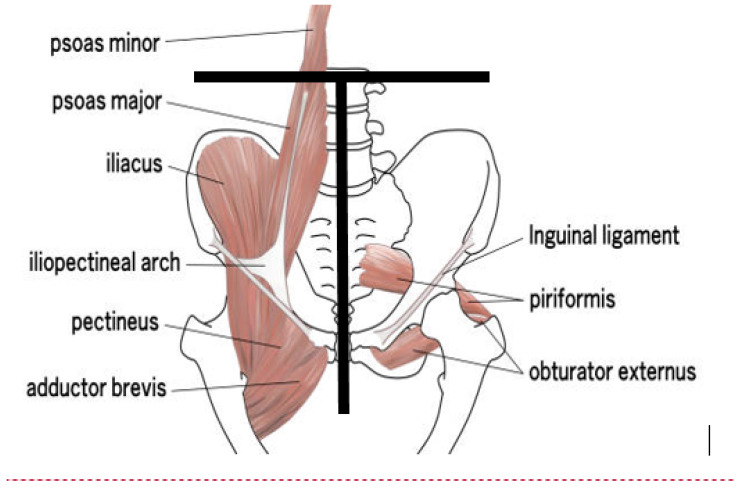
Additional muscle mass included in new ALMI.

Indeed, using new landmarks, the merging should allow for greater reliability of the ALMI and include the gluteal and pelvi-trochanteric muscles in the ALMI (Figure 2).

#### 2.2.3. Scan Analysis

The scans were analyzed by three different operators, each with varying levels of experience in DEXA analysis. Indeed, manual delineation of the ROIs is described as a source of variability. At our university hospital, we had the opportunity to test this source of variability by comparing the reproducibility of measurements between two experienced investigators (a rheumatologist with 6 years of experience and DEXA consultant within the department; a sports medicine physician with 3 years of experience) and a sports physician inexperienced in DEXA. The inexperienced investigator was completing an internship in physical medicine and rehabilitation, with a specialization in sports medicine. He had been working in the department for one month at the time of subject inclusion and had not previously had the opportunity to perform DEXA assessments. Due to the study objectives, we did not train him in DEXA procedures as we usually do. We simply provided him with written information, without practical training on delineating regions of interest.

An initial automated analysis (QDR software for Windows version 12.4; Hologic, Waltham, MA, USA) was performed as a reference. Each physician then performed the measurement using both methods, blinded to the other two physicians.

The following written explanations were provided to the inexperienced sports medicine physician:

- “Conventional measure: The line separating the head should pass under the mandible. The line separating the spine from the thorax should pass along the spine. The line separating the upper limbs from the body should pass by the joint gleno-humeral. The line separating the pelvis from the trunk should pass over the upper edge of the iliac crests. The line separating the pelvis from the lower limbs should cross by the middle of the neck of the femur.”
- “New measure: The line separating the head should pass under the mandible. The line separating the spine from the thorax should pass along the spine. The line separating the upper limbs from the body should pass by the joint gleno-humeral. The line separating the pelvis from the trunk should pass over the upper edge of the iliac crests. The point of intersection of the lines separating the pelvis from the lower limbs should put on the middle of the line separating the pelvis from the trunk.”

The DXA measurements included whole-body measurements of DXA mass (kg), absolute fat mass (FM) (kg), percentage FM (% FM), fat-free mass (FFM) (kg), bone mineral content (BMC) (kg) and bone mineral density (BMD) (kg.m^−2^), and lean mass (LM) (kg) defined as FFM minus BMC. All these parameters are assessed for each ROI. The sum of the lean masses of the limbs (upper and lower) constitutes the appendicular lean mass (ALM), which is indexed by the squared height to define the appendicular lean mass index (ALMI) (kg.m^−2^).

#### 2.2.4. Statistical Analysis

We compared the reproducibility between automated and conventional three-operator analysis of ALMI. Inter-operator reproducibility of ALMI, based on percentage variation, was compared between the conventional method and the new method.

Concordance between methods was assessed using a Bland–Altman multi-operator adjustment and limits of agreement with a 95% confidence interval [19]. This adaptation of Bland–Altman plots presents the mean ALMI measurements on the *x*-axis, while the *y*-axis represents the difference in measurements between each rater’s measure and the mean for all three raters. A scatter plot was used to illustrate the relationship between the paired method differences and the mean of the two methods.

A two-way analysis of variance (ANOVA) was performed, as well as a one-way ANOVA for each method. Mean inter-operator differences were calculated as the average of the absolute differences between each pair of raters for the same subject, representing the mean difference observed between two measurements from different raters.

Intraclass correlation coefficients (ICCs) were calculated using a two-way random model for single raters, with agreement as the primary outcome (ICC (A,1)) and reliability as the secondary outcome (ICC (C,1)). The generalized confidence interval method was used to calculate the 95% confidence interval limits of the ICC [21]. The choice of the model relied on the intent to estimate population’s ICC for any new rater, based on the same set of raters, but without the random choice of this set. The choice of absolute agreement over consistency is justified by the need to differentiate players based on a single measurement, potentially performed by different evaluators. This approach allows for monitor developments across different centers and, ultimately, for making training or clinical decisions based on these measurements. The ANOVA results enable the calculation of other ICC values.

Statistical analysis was performed with R v.4.3.1 (R Core Team (2023). R: A Language and Environment for Statistical Computing. R Foundation for Statistical Computing, Vienna, Austria). The data are presented as means ± (SD), and the statistical significance was set at *p* < 0.05.

## 3. Results

### 3.1. Population Characteristics

The twenty subjects were males, with an age of 25.9 (SD: 4.9) years, a weight of 79.4 kg (9.4), and a height of 1.83 m (0.09). Their body mass index was 23.6 kg/m^2^ (1.1). Average whole-body lean mass was 63.82 kg (6.74), whole-body fat mass 10.87 kg (2.29), and whole-body bone mineral content 3.65 kg (0.47). When indexed to the body surface average whole-body lean mass was 19.05 kg/m^2^ (0.75), whole-body fat mass 3.2 kg/m^2^ (0.5), and whole-body bone mineral content equivalent to whole-body bone mineral density 1.48 kg/m^2^ (0.10). The average weight of the lower limbs was 14.4 kg (2.0), with an average fat-free mass of 12.4 kg (1.5) and an average percentage of fat 13% (2.0). There was a 4% difference between the two legs in fat mass, lean mass, and total mass. The average weight of the upper limbs was 4.6 kg (0.7), with an average fat-free mass of 4.0 kg (0.6) and an average percentage of fat 13% (2.0). There was a 7% difference between the two legs in fat mass, 1% in lean mass, and 0% in total mass.

### 3.2. Appendicular Lean Mass Index (ALMI) Using the Standard Method

Using automatic analysis, the appendicular lean mass index (ALMI) was 9.20 kg/m^2^ (0.67), measured with the standard method. ALMI values were 9.28 kg/m^2^ (0.62), 9.20 kg/m^2^ (0.65) and 9.13 kg/m^2^ (0.64) when measured by the first, second, and third operator, respectively, using the standard analysis (Table 1). The first operator performed the acquisition (JB), and then three operators conducted the analysis using the two methods (JB operator 1, CE operator 2, and EF operator 3). There was no significant bias detected by ANOVA (Table 2). Mean inter-operator difference was 0.099 kg/m^2^ (0.06), 1.08% (0.38).

### 3.3. Appendicular Lean Mass Index (ALMI) Using the New Method

Using the new method to analyze the appendicular lean mass index (ALMI), the first operator measured an ALMI of 11.90 kg/m^2^ (0.66). The second operator found an ALMI of 11.84 kg/m^2^ (0.63), and the third operator measured an ALMI of 11.83 kg/m^2^ (0.65), without significant bias detected. Mean inter-operator difference with the new method was 0.047 kg/m^2^ (0.03), 0.39% (0.22) (Table 1).

### 3.4. Comparison Between Conventional and Strasbourg Method

The same three raters evaluated each subject using the two methods. This resulted in 120 measurements, with three measurements per subject. There was a significant difference between the means of the two methods (*p* < 0.001).

**Figure 3:**
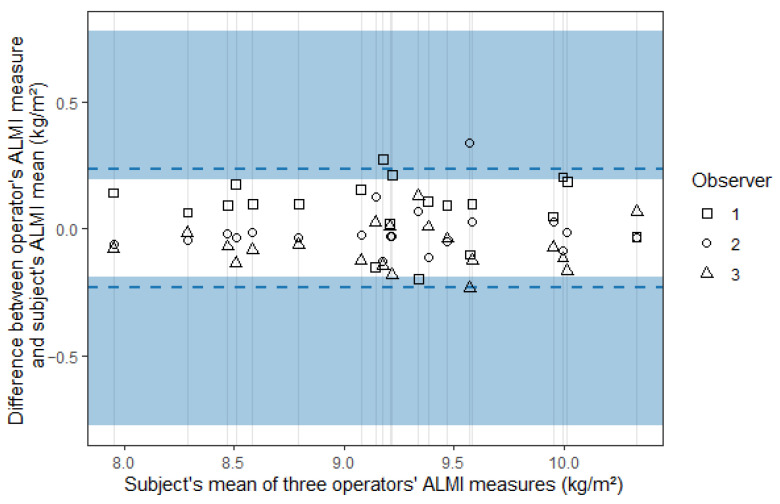
Agreement plot for the appendicular lean mass index (ALMI) measured using the standard method.

**Figure 4:**
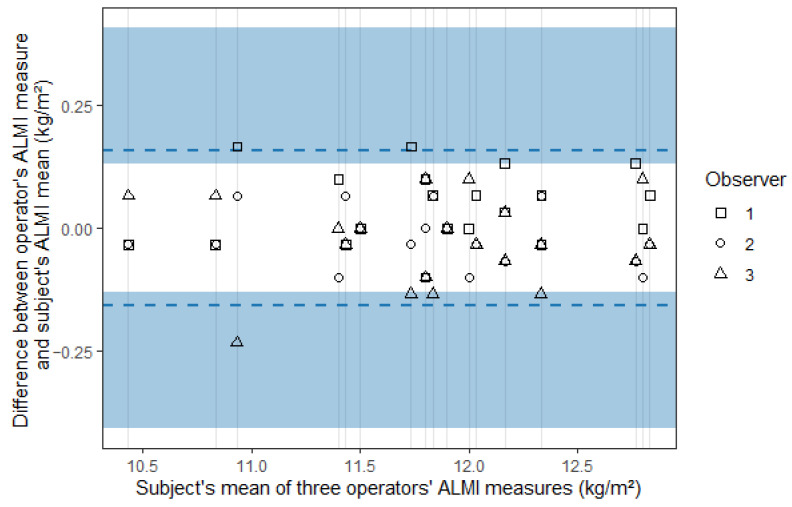
Agreement plot for the appendicular lean mass index (ALMI) measured using the new method.

The limits of agreement were ±0.232 [0.193; 0.776] and ±0.157 [0.131; 0.408] for the standard and the new method, respectively, and agreement plots are presented in Figure 3 and Figure 4, where the blue areas represent the 95% confidence intervals.

The ICC (A,1) was 0.949 [0.881; 0.979] for the standard method and 0.977 [0.951; 0.990] for the new method. The ICC (C,1) was 0.960 [0.918; 0.983] and 0.979 [0.957; 0.991] for the standard and the new method, respectively, (Table 3).

## 4. Discussion

The aim of this study was to determine the feasibility of new scan line adjustment during DEXA to include greater gluteal and adductor muscle mass in professional soccer players. We also examined inter-operator reproducibility compared to the standard method. The main results show that both new and conventional methods demonstrated excellent reproducibility. However, reproducibility and inter-operator variability were better with the new scan line adjustment. Furthermore, the inclusion of greater muscle mass in the gluteal and adductors was easy to perform.

### 4.1. Feasibility and Reproducibility of Standard and New DEXA Methods

DEXA imaging and analysis are operator-dependent and rely heavily on precise positioning of the studied subject and manual delineation of regions of interest [17,23,24,27,30,31,32,33]. This remains true when a new approach is proposed, which should allow similar or even superior feasibility in terms of scan line positioning and enable longitudinal follow-up [45].

Regarding the standard method, the positioning of the acquisition lines, particularly those passing through the center of the femoral neck, can be a source of intra-operator variability. The angle between the horizontal of the pelvis and the mid-pelvis-leg line varies according to the position of the center of the femoral neck. The opening or closing of this angle results in the inclusion of more or less adductor and gluteal mass. This positioning can be a source of interpretation for the operator, both in locating the center of the neck and defining the angle of the line. This is due to the absence of a common landmark such as a bony prominence or joint space. Despite this limitation, this method has shown excellent reproducibility thanks to ICC calculations.

Regarding the new method, reproducibility was also excellent, with an even narrower range with this new approach. The 95% confidence interval of the limits of agreement also showed that the new method could be more reproducible, as it included lower values. To avoid some pitfalls of the standard method, well-defined anatomical structures were used to place new landmarks, namely the top of the iliac crests for a horizontal line (delimitation of the trunk and lower limbs) and the vertical line passing through the pubic syndesmosis (delimitation of the two lower limbs). This approach, based on structures present in all patients, should enable a reduction in potential interpretation of the line positioning.

In this view, magnetic resonance imaging (MRI), with its precise delineation of muscles, could confirm the anatomical rationale for our approach. Indeed, MRI has demonstrated the ability to analyze muscle mass from different aspects with precision and a strong correlation with DEXA for the muscle mass assessment [46,47].

### 4.2. Probable Perspectives Opened up by the Assessment of the Pelvi-Trochanteric Muscles

The aim of the new method was to include muscles such as the gluteus and adductors, which are widely involved in sports performance and muscle injuries in soccer players [38,48,49]. Indeed, the gluteus maximus contributes to hip extension and propulsion during sprinting and jumping and improves explosive power, vertical jump height, and postural control. Accordingly, interventional studies show significant improvements in jumping and sprinting parameters after gluteus maximus-focused training [50,51,52]. The gluteus medius and minimus stabilize the pelvis in the frontal plane, control dynamic valgus at the knee, and contribute to balance during one-legged tasks and landing. Their higher activation is correlated with better joint alignment and reduced injury risk [53,54]. The adductor muscle group provides medial stabilization of the thigh, which is crucial for direction changes, kicking, and sprint mechanics. They play a key role in football-specific movements and injury resilience [55,56].

Indeed, acute adductor injuries are common. The overall incidence of adductor strains was 1.29 injuries per 1000 exposures across 25 college sports, with men’s soccer having the highest incidences (3.15 per 1000) [57].

The hip abductor muscles, including the gluteus medius, minimus, and the tensor fasciae latae, stabilize the pelvis and hip during weight-bearing activities. It is recognized that the gluteus medius and minimus are heavily engaged during the stance and swing phases of running, with an electromyographic amplitude 3 to 8 times greater than that of walking and a peak amplitude during the stance phase [58,59].

Therefore, groin injuries and pains are common in football and more generally in sport activities, ranging from 7% to 18% [60]. During a season, 21% of professional football players suffer a groin injury [61]. Interestingly, gluteal muscle weakness and abnormal hip mechanics are associated with lower limb injury [62]. Therefore, unilateral strength imbalances, as well as bilateral or inter-leg asymmetry of lower limb strength, have been identified [63] as the cause of several injuries, particularly hamstring strains. On the other hand, bilateral asymmetry of less than 5% suggests good muscle condition [64], which may reduce muscle discomfort and subsequent hamstring injuries [65].

Thus, this assessment of lower limb differences constitutes an important screening tool for injury risk and a valuable reference for prescribing rehabilitation programs [66,67]. Indeed, adductor’s injuries have high recurrence rates in professional footballers.

For injury prevention and monitoring, our method appears more reproducible and allows the precise identification of muscle asymmetries requiring a dedicated rehabilitation program. Combined with an assessment of hip strength by isokinetic dynamometry [68,69,70], better monitoring of athletes in terms of injury prevention, recovery, and performance could therefore be proposed [43,45].

Indeed, in professional football, one of the criteria for a return to play after a hamstring injury is the normalization of the isokinetic strength assessment between the two lower limbs [71]. It has recently been shown that there is a strong correlation between DEXA composition balance and isokinetic strength balance [44]. Moreover, better results in the surgical management of anterior cruciate ligament rupture are obtained according to body composition analyzed by DEXA [72]. In view of these recent data, body composition assessment, particularly the comparison between limbs including all the muscle masses involved in movement, as is the case with isokinetic assessments, could be included in the decision to return to play. Thus, knowledge of pre-injury muscle mass could provide a target for enabling return-to-play decisions, allowing for an accurate characterization of the muscular status of footballers [45].

There are several limitations to consider in this study. One of them is the small number of subjects included and the absence of a sample size calculation, which may impact the interpretation of our findings. However, our sample size, although relatively small, allowed us to conclude that both techniques have excellent reproducibility, with better-observed sample ICC for the new technique. A similar result was also observed in another study investigating fifteen subjects [59]. Second, although our population is homogeneous (gender, sport practiced, and level of training), confirmation data are needed before generalization. Indeed, to make the findings generalizable to many situations, it would be necessary to study more subjects, ranging from healthy subjects to elderly people at high risk of falling, including athletes practicing different endurance or resistance sports. In this regard, our data providing accurate variances estimation could also be used to design future studies on larger cohorts with different sports and levels of training. This will also challenge the clinical relevance of measuring additional adductor and gluteal muscle mass.

## 5. Conclusions

In conclusion, the inclusion of greater gluteal and adductors muscle mass in DEXA measurements is easy to achieve. This new method, using a novel scan line adjustment, allows for better reproducibility and reduced inter-operator variability compared to the standard approach. Therefore, although the involvement of these muscles in football players’ performance and injuries was not explored in our study, and although it requires further testing, our data support longitudinal DEXA monitoring on a larger cohort of athletes to determine whether it could contribute to the early diagnosis of abnormalities and thus prevent muscle injuries and protect athletes’ health.

## Figures and Tables

**Table 1:** Description of ALMI values by method and operator.

	Total	Operators		
		1	2	3
**Total (N = 120) †**	10.528 (1.47)	10.588 (1.47)	10.516 (1.48)	10.479 (1.50)
**Standard method (N = 60) †**	9.203 (0.63)	9.281 (0.62)	9.196 (0.65)	9.132 (0.64)
**New method (N = 60) †**	11.852 (0.64)	11.895 (0.66)	11.835 (0.63)	11.825 (0.65)

† Mean (standard deviation)

**Table 2:** Two-way and one-way analysis of variance.

	Degrees of Freedom	Sum of Squares	Mean Squares	F Value	Pr (>F)
**Total (N = 120)**					
**Method**	1	210.410	210.410	509.518	***<0.001***
**Operator**	2	0.247	0.123	0.299	0.742
**Method: operator**	2	0.032	0.016	0.039	0.962
**Residuals**	114	47.077	0.413		
**Standard method (N = 60)**					
**Operator**	2	0.222	0.111	0.272	0.763
**Residuals**	57	23.245	0.408		
**New method (N = 60)**					
**Operator**	2	0.057	0.029	0.069	0.934
**Residuals**	57	23.832	0.418		

**Table 3:** ICC and 95% confidence interval for agreement and consistency of the two methods.

Method	Type	ICC	95% CI
Standard	Agreement	0.949	[0.881; 0.979]
	Consistency	0.960	[0.918; 0.983]
New method	Agreement	0.977	[0.951; 0.990]
	Consistency	0.979	[0.957; 0.991]

## References

[1] Campa F., Toselli S., Mazzilli M., Gobbo L.A., Coratella G. (2021). Assessment of Body Composition in Athletes: A Narrative Review of Available Methods with Special Reference to Quantitative and Qualitative Bioimpedance Analysis. Nutrients.

[2] Hind K., Slater G., Oldroyd B., Lees M., Thurlow S., Barlow M., Shepherd J. (2018). Interpretation of Dual-Energy X-Ray Absorptiometry-Derived Body Composition Change in Athletes: A Review and Recommendations for Best Practice. J. Clin. Densitom..

[3] Bilsborough J.C., Greenway K., Opar D., Livingstone S., Cordy J., Coutts A.J. (2014). The Accuracy and Precision of DXA for Assessing Body Composition in Team Sport Athletes. J. Sports Sci..

[4] Santos D.A., Dawson J.A., Matias C.N., Rocha P.M., Minderico C.S., Allison D.B., Sardinha L.B., Silva A.M. (2014). Reference Values for Body Composition and Anthropometric Measurements in Athletes. PLoS ONE.

[5] Shepherd J.A., Ng B.K., Sommer M.J., Heymsfield S.B. (2017). Body Composition by DXA. Bone.

[6] Milanese C., Cavedon V., Corradini G., De Vita F., Zancanaro C. (2015). Seasonal DXA-Measured Body Composition Changes in Professional Male Soccer Players. J. Sports Sci..

[7] Ramírez-Munera M., Arcusa R., López-Román F.J., Victoria-Montesinos D., García-Muñoz A.M., Ávila-Gandía V., Pérez-Piñero S., Marhuenda J. (2024). Anthropometric and Body Composition Changes during Pre-Season of Spanish Professional Female Soccer Players According to Playing Position. Nutrients.

[8] Melin A.K., Heikura I.A., Tenforde A., Mountjoy M. (2019). Energy Availability in Athletics: Health, Performance, and Physique. Int. J. Sport Nutr. Exerc. Metab..

[9] Aragon A.A., Schoenfeld B.J., Wildman R., Kleiner S., VanDusseldorp T., Taylor L., Earnest C.P., Arciero P.J., Wilborn C., Kalman D.S. (2017). International Society of Sports Nutrition Position Stand: Diets and Body Composition. J. Int. Soc. Sports Nutr..

[10] Maughan R.J., Internationales Olympisches Komitee (2009). The Encyclopaedia of Sports Medicine in Collabororation with the International Federation of Sports Medicine: An IOC Medical Commission Publication. Olympic Textbook of Science in Sport.

[11] Genton L., Mareschal J., Karsegard V.L., Achamrah N., Delsoglio M., Pichard C., Graf C., Herrmann F.R. (2019). An Increase in Fat Mass Index Predicts a Deterioration of Running Speed. Nutrients.

[12] Dopsaj M., Zuoziene I.J., Milić R., Cherepov E., Erlikh V., Masiulis N., di Nino A., Vodičar J. (2020). Body Composition in International Sprint Swimmers: Are There Any Relations with Performance?. Int. J. Environ. Res. Public Health.

[13] Bell D.R., Sanfilippo J.L., Binkley N., Heiderscheit B.C. (2014). Lean Mass Asymmetry Influences Force and Power Asymmetry during Jumping in Collegiate Athletes. J. Strength Cond. Res..

[14] Toomey C.M., Whittaker J.L., Nettel-Aguirre A., Reimer R.A., Woodhouse L.J., Ghali B., Doyle-Baker P.K., Emery C.A. (2017). Higher Fat Mass Is Associated with a History of Knee Injury in Youth Sport. J. Orthop. Sports Phys. Ther..

[15] Rueda-Cordoba M., Martin-Olmedo J.J., Espinar S., Ruiz J.R., Jurado-Fasoli L. (2025). Multidimensional Differences Between Athletes of Endurance, Strength, and Intermittent Sports: Body Composition, Diet, Resting Metabolic Rate, Physical Activity, Sleep Quality, and Subjective Well-Being. Nutrients.

[16] Al-Hayani A. (2009). The Functional Anatomy of Hip Abductors. Folia Morphol..

[17] Bazzocchi A., Ponti F., Albisinni U., Battista G., Guglielmi G. (2016). DXA: Technical Aspects and Application. Eur. J. Radiol..

[18] Burén J., Svensson M., Liv P., Sjödin A. (2024). Effects of a Ketogenic Diet on Body Composition in Healthy, Young, Normal-Weight Women: A Randomized Controlled Feeding Trial. Nutrients.

[19] Christensen H.S., Borgbjerg J., Børty L., Bøgsted M. (2020). On Jones et al.’s Method for Extending Bland-Altman Plots to Limits of Agreement with the Mean for Multiple Observers. BMC Med. Res. Methodol..

[20] Giannini M., Charles A.-L., Evrard C., Blaess J., Bouchard-Marmen M., Debrut L., Perniola S., Laverny G., Javier R.-M., Charloux A. (2024). Sarcopenia Assessed by DXA and Hand-Grip Dynamometer: A Potential Marker of Damage, Disability and Myokines Imbalance in Inflammatory Myopathies. Rheumatology.

[21] Ionan A.C., Polley M.-Y.C., McShane L.M., Dobbin K.K. (2014). Comparison of Confidence Interval Methods for an Intra-Class Correlation Coefficient (ICC). BMC Med. Res. Methodol..

[22] Levy D., Giannini M., Oulehri W., Riou M., Marcot C., Pizzimenti M., Debrut L., Charloux A., Geny B., Meyer A. (2022). Long Term Follow-Up of Sarcopenia and Malnutrition after Hospitalization for COVID-19 in Conventional or Intensive Care Units. Nutrients.

[23] Libber J., Binkley N., Krueger D. (2012). Clinical Observations in Total Body DXA: Technical Aspects of Positioning and Analysis. J. Clin. Densitom..

[24] Lohman M., Tallroth K., Kettunen J.A., Marttinen M.T. (2009). Reproducibility of Dual-Energy X-Ray Absorptiometry Total and Regional Body Composition Measurements Using Different Scanning Positions and Definitions of Regions. Metabolism.

[25] Lukaski H., Raymond-Pope C.J. (2021). New Frontiers of Body Composition in Sport. Int. J. Sports Med..

[26] Nana A., Slater G.J., Stewart A.D., Burke L.M. (2015). Methodology Review: Using Dual-Energy X-Ray Absorptiometry (DXA) for the Assessment of Body Composition in Athletes and Active People. Int. J. Sport Nutr. Exerc. Metab..

[27] Pizzimenti M., Meyer A., Charles A.L., Giannini M., Chakfé N., Lejay A., Geny B. (2020). Sarcopenia and Peripheral Arterial Disease: A Systematic Review. J. Cachexia Sarcopenia Muscle.

[28] Valenzano A.A., Vasco P., D’Orsi G., Marzovillo R.R.R., Torquato M., Messina G., Polito R., Cibelli G. (2025). Influence of Intermittent Fasting on Body Composition, Physical Performance, and the Orexinergic System in Postmenopausal Women: A Pilot Study. Nutrients.

[29] Lorente Ramos R.M., Armán J.A., Galeano N.A., Hernández A.M., García Gómez J.M., Molinero J.G. (2012). Dual Energy X-Ray Absorptimetry: Fundamentals, Methodology, and Clinical Applications. Radiologia.

[30] Baim S., Binkley N., Bilezikian J.P., Kendler D.L., Hans D.B., Lewiecki E.M., Silverman S. (2008). Official Positions of the International Society for Clinical Densitometry and Executive Summary of the 2007 ISCD Position Development Conference. J. Clin. Densitom..

[31] Collins J., Maughan R.J., Gleeson M., Bilsborough J., Jeukendrup A., Morton J.P., Phillips S.M., Armstrong L., Burke L.M., Close G.L. (2021). UEFA Expert Group Statement on Nutrition in Elite Football. Current Evidence to Inform Practical Recommendations and Guide Future Research. Br. J. Sports Med..

[32] Dallman J., Herda A., Cleary C.J., Morey T., Diederich A., Vopat B.G., Vopat L.M. (2024). A Brief Review of the Literature for Published Dual-Energy X-Ray Absorptiometry Protocols for Athletes. Sports Health.

[33] Ackerman K.E., Rogers M.A., Heikura I.A., Burke L.M., Stellingwerff T., Hackney A.C., Verhagen E., Schley S., Saville G.H., Mountjoy M. (2023). Methodology for Studying Relative Energy Deficiency in Sport (REDs): A Narrative Review by a Subgroup of the International Olympic Committee (IOC) Consensus on REDs. Br. J. Sports Med..

[34] Poltronieri T.S., de Paula N.S., Chaves G.V. (2020). Assessing Skeletal Muscle Radiodensity by Computed Tomography: An Integrative Review of the Applied Methodologies. Clin. Physiol. Funct. Imaging.

[35] Bellinger P., Bourne M.N., Duhig S., Lievens E., Kennedy B., Martin A., Cooper C., Tredrea M., Rice H., Derave W. (2021). Relationships between Lower Limb Muscle Characteristics and Force–Velocity Profiles Derived during Sprinting and Jumping. Med. Sci. Sports Exerc..

[36] Yoshimoto T., Chiba Y., Ohnuma H., Sugisaki N. (2025). Investigation of Trunk and Pelvis Muscle Activity during Sprinting Using T2-Weighted Magnetic Resonance Imaging. J. Hum. Kinet..

[37] Kamina P. (2009). Anatomie Clinique.

[38] Hughes P.E., Hsu J.C., Matava M.J. (2002). Hip Anatomy and Biomechanics in the Athlete. Sports Med. Arthrosc. Rev..

[39] Watanabe K., Nunome H., Inoue K., Iga T., Akima H. (2020). Electromyographic Analysis of Hip Adductor Muscles in Soccer Instep and Side-Foot Kicking. Sports Biomech..

[40] Erlandson M.C., Lorbergs A.L., Mathur S., Cheung A.M. (2016). Muscle Analysis Using pQCT, DXA and MRI. Eur. J. Radiol..

[41] Tanaka M., Kanayama M., Oha F., Shimamura Y., Tsujimoto T., Hasegawa Y., Hashimoto T., Nojiri H., Ishijima M. (2023). Potential of Whole-Body Dual-Energy X-Ray Absorptiometry to Predict Muscle Size of Psoas Major, Gluteus Maximus and Back Muscles. BMC Musculoskelet. Disord..

[42] Neumann D.A. (2002). Kinesiology of the Musculoskeletal System: Foundations for Physical Rehabilitation.

[43] Junge A., Dvořák J. (2015). Football Injuries during the 2014 FIFA World Cup. Br. J. Sports Med..

[44] Cataldi D., Bennett J.P., Quon B.K., Leong L., Kelly T.L., Binder A.M., Evans W.J., Prado C.M., Heymsfield S.B., Shepherd J.A. (2025). Association of Body Composition Measures to Muscle Strength Using DXA, D3Cr, and BIA in Collegiate Athletes. Sci. Rep..

[45] Rodríguez J.L.E., García J.R., Jiménez-Rubio S. (2024). Return to Performance of a Soccer Player with an Adductor Longus Injury: A Case Report. Medicina.

[46] Borga M., West J., Bell J.D., Harvey N.C., Romu T., Heymsfield S.B., Leinhard O.D. (2018). Advanced Body Composition Assessment: From Body Mass Index to Body Composition Profiling. J. Investig. Med..

[47] Hooijmans M.T., Schlaffke L., Bolsterlee B., Schlaeger S., Marty B., Mazzoli V. (2024). Compositional and Functional MRI of Skeletal Muscle: A Review. J. Magn. Reson. Imaging.

[48] Thorborg K., Serner A., Petersen J., Madsen T.M., Magnusson P., Hölmich P. (2011). Hip Adduction and Abduction Strength Profiles in Elite Soccer Players: Implications for Clinical Evaluation of Hip Adductor Muscle Recovery after Injury. Am. J. Sports Med..

[49] Dupré T., Potthast W. (2024). Are Sprint Accelerations Related to Groin Injuries? A Biomechanical Analysis of Adolescent Soccer Players. Sports Biomech..

[50] Gallego-Izquierdo T., Vidal-Aragón G., Calderón-Corrales P., Acuña Á., Achalandabaso-Ochoa A., Aibar-Almazán A., Martínez-Amat A., Pecos-Martín D. (2020). Effects of a Gluteal Muscles Specific Exercise Program on the Vertical Jump. Int. J. Environ. Res. Public Health.

[51] Omi Y., Sugimoto D., Kuriyama S., Kurihara T., Miyamoto K., Yun S., Kawashima T., Hirose N. (2018). Effect of Hip-Focused Injury Prevention Training for Anterior Cruciate Ligament Injury Reduction in Female Basketball Players: A 12-Year Prospective Intervention Study. Am. J. Sports Med..

[52] Collings T.J., Bourne M.N., Barrett R.S., Meinders E., Gonçalves B.A.M., Shield A.J., Diamond L.E. (2023). Gluteal Muscle Forces during Hip-Focused Injury Prevention and Rehabilitation Exercises. Med. Sci. Sports Exerc..

[53] Nascimento M.B., Vilarinho L.G., Lobato D.F.M., Dionisio V.C. (2023). Role of Gluteus Maximus and Medius Activation in the Lower Limb Biomechanical Control during Functional Single-Leg Tasks: A Systematic Review. Knee.

[54] Ceballos-Laita L., Carrasco-Uribarren A., Cabanillas-Barea S., Pérez-Guillén S., Medrano-de-la-Fuente R., Hernando-Garijo I., Jiménez-del-Barrio S. (2022). Relationship between Hip Abductor Muscle Strength and Frontal Plane Kinematics: A Cross-Sectional Study in Elite Handball Athletes. Appl. Sci..

[55] Charnock B.L., Lewis C.L., Garrett W.E., Queen R.M. (2009). Adductor Longus Mechanics during the Maximal Effort Soccer Kick. Sports Biomech..

[56] Ibrahim A., Murrell G.A.C., Knapman P. (2007). Adductor Strain and Hip Range of Movement in Male Professional Soccer Players. J. Orthop. Surg..

[57] McHugh M.P., Nicholas S.J., Tyler T.F. (2023). Adductor Strains in Athletes. Int. J. Sports Phys. Ther..

[58] Semciw A., Neate R., Pizzari T. (2016). Running Related Gluteus Medius Function in Health and Injury: A Systematic Review with Meta-Analysis. J. Electromyogr. Kinesiol..

[59] Nunes G.S., Pizzari T., Neate R., Barton C.J., Semciw A. (2020). Gluteal Muscle Activity during Running in Asymptomatic People. Gait Posture.

[60] Waldén M., Hägglund M., Ekstrand J. (2015). The Epidemiology of Groin Injury in Senior Football: A Systematic Review of Prospective Studies. Br. J. Sports Med..

[61] Mosler A.B., Weir A., Eirale C., Farooq A., Thorborg K., Whiteley R.J., Hölmich P., Crossley K.M. (2018). Epidemiology of Time Loss Groin Injuries in a Men’s Professional Football League: A 2-Year Prospective Study of 17 Clubs and 606 Players. Br. J. Sports Med..

[62] Powers C.M. (2010). The Influence of Abnormal Hip Mechanics on Knee Injury: A Biomechanical Perspective. J. Orthop. Sports Phys. Ther..

[63] Burkett L.N. (1970). Causative Factors in Hamstring Strains. Med. Sci. Sports.

[64] Croisier J.L., Crielaard J.M. (2000). Hamstring Muscle Tear with Recurrent Complaints: An Isokinetic Profile. Isokinet. Exerc. Sci..

[65] Croisier J.L., Ganteaume S., Binet J., Genty M., Ferret J.-M. (2008). Strength Imbalances and Prevention of Hamstring Injury in Professional Soccer Players: A Prospective Study. Am. J. Sports Med..

[66] Cheung R.T.H., Smith A.W., Wong D.P. (2012). H:Q Ratios and Bilateral Leg Strength in College Field and Court Sports Players. J. Hum. Kinet..

[67] Carvalho A., Brown S., Abade E. (2016). Evaluating Injury Risk in First and Second League Professional Portuguese Soccer: Muscular Strength and Asymmetry. J. Hum. Kinet..

[68] Moreno-Pérez V., Beato M., Del Coso J., Hernández-Davó J.L., Soler A., Peñaranda-Moraga M., Madruga-Parera M., Romero-Rodríguez D. (2022). Intra and Inter-Tester Reliability of a Novel Device to Assess Gluteal Muscle Strength in Professional Football Players. Res. Sports Med..

[69] Widler K.S., Glatthorn J.F., Bizzini M., Impellizzeri F.M., Munzinger U., Leunig M., Maffiuletti N.A. (2009). Assessment of Hip Abductor Muscle Strength. A Validity and Reliability Study. J. Bone Jt. Surg..

[70] Vaillancourt N., Montpetit C., Carile V., Fortin M. (2024). DEXA Body Composition Asymmetry Analysis and Association to Injury Risk and Low Back Pain in University Soccer Players. Int. J. Environ. Res. Public Health.

[71] Delvaux F., Rochcongar P., Bruyère O., Bourlet G., Daniel C., Diverse P., Reginster J.-Y., Croisier J.-L. (2013). Return-to-Play Criteria after Hamstring Injury: Actual Medicine Practice in Professional Soccer Teams. Br. J. Sports Med..

[72] Buck A.N., Moore S.R., Smith-Ryan A.E., Schwartz T.A., Nelson A.E., Davis-Wilson H., Blackburn J.T., Pietrosimone B. (2025). Body Composition, Not Body Mass Index, Is Associated with Clinical Outcomes Following ACL Reconstruction. Med. Sci. Sports Exerc..

